# Genome-Wide Search Identifies 1.9 Mb from the Polar Bear Y Chromosome for Evolutionary Analyses

**DOI:** 10.1093/gbe/evv103

**Published:** 2015-05-27

**Authors:** Tobias Bidon, Nancy Schreck, Frank Hailer, Maria A. Nilsson, Axel Janke

**Affiliations:** ^1^Senckenberg Biodiversity and Climate Research Centre Frankfurt, Frankfurt am Main, Germany; ^2^International Graduate School of Science and Engineering (IGSSE), Technische Universität München, Garching, Germany; ^3^School of Biosciences, Cardiff University, Wales, United Kingdom; ^4^Institute for Ecology, Evolution & Diversity, Goethe University Frankfurt, Germany

**Keywords:** sex chromosome, patriline, male inheritance, Ursidae, Y chromosome, divergence

## Abstract

The male-inherited Y chromosome is the major haploid fraction of the mammalian genome, rendering Y-linked sequences an indispensable resource for evolutionary research. However, despite recent large-scale genome sequencing approaches, only a handful of Y chromosome sequences have been characterized to date, mainly in model organisms. Using polar bear (*Ursus maritimus*) genomes, we compare two different in silico approaches to identify Y-linked sequences: 1) Similarity to known Y-linked genes and 2) difference in the average read depth of autosomal versus sex chromosomal scaffolds. Specifically, we mapped available genomic sequencing short reads from a male and a female polar bear against the reference genome and identify 112 Y-chromosomal scaffolds with a combined length of 1.9 Mb. We verified the in silico findings for the longer polar bear scaffolds by male-specific in vitro amplification, demonstrating the reliability of the average read depth approach. The obtained Y chromosome sequences contain protein-coding sequences, single nucleotide polymorphisms, microsatellites, and transposable elements that are useful for evolutionary studies. A high-resolution phylogeny of the polar bear patriline shows two highly divergent Y chromosome lineages, obtained from analysis of the identified Y scaffolds in 12 previously published male polar bear genomes. Moreover, we find evidence of gene conversion among *ZFX* and *ZFY* sequences in the giant panda lineage and in the ancestor of ursine and tremarctine bears. Thus, the identification of Y-linked scaffold sequences from unordered genome sequences yields valuable data to infer phylogenomic and population-genomic patterns in bears.

## Introduction

Genomic sequence data have become an important resource for evolutionary biology, and new sequenced genomes are becoming available at increasing speed. The mammalian genome consists of autosomes, sex chromosomes and mitochondrial (mt) DNA, which are differentially inherited. These parts of the genome can thus provide information about distinctive aspects of a species’ evolutionary history (Chesser and Baker 1996; Veeramah and Hammer 2014).

For technical reasons, the maternally inherited mtDNA has been a standard tool to study evolutionary processes in model and nonmodel organisms (Wilson et al. 1985). Consequently, the first available genomic resources for evolutionary studies were fully sequenced mt genomes (Anderson et al. 1981; Janke et al. 1994). The paternally inherited counterpart of mtDNA is the male-specific Y chromosome, one of two sex chromosomes in the mammalian genome. Similar to mtDNA, the Y chromosome is haploid, lacks interchromosomal recombination for most of its length, and is uniparentally inherited. These properties allow the inference of long and high-resolution haplotypes, enabling researchers to trace the evolutionary history of male lineages over time (Jobling and Tyler-Smith 2003; Wei et al. 2013). Biparentally inherited autosomes provide the largest amount of sequence data, but their phylogenetic analysis can be complicated by reticulate evolution (Posada et al. 2002).

Polar bears have recently been in the focus of genome scale evolutionary analyses and genomic sequences have been used to address the evolution, population history, and unique adaptations of this high arctic mammal (Hailer et al. 2012; Miller et al. 2012; Cahill et al. 2013, 2015; Cronin et al. 2014; Liu et al. 2014). Furthermore, short Y-chromosomal sequences and six Y-linked microsatellites in polar and brown bears were used to investigate the distribution of male-specific genetic variation across their ranges (Bidon et al. 2014). The application of Y-chromosomal markers is particularly interesting in many mammals, because sex-specific differences in dispersal behavior are predicted to affect phylogeographic and population genetic conclusions that have so far been drawn almost exclusively from female-inherited mtDNA.

Y-chromosomal sequences are important in studies of evolutionary history, chromosome structure, and forensic applications (Jobling and Tyler-Smith 2003; Kayser 2007; Hallast et al. 2013). The Y chromosome’s unique evolutionary viewpoint has been used to investigate patterns of domestication and migration, for example, in horses and dogs (Sacks et al. 2013; Wallner et al. 2013), and to study human phylogeography and migration (Wei et al. 2013; Scozzari et al. 2014; Van Oven et al. 2014).

Despite the wealth of genomic data, identification of large amounts of Y-chromosomal sequences from high throughput sequencing data is rarely done. Genome sequences are usually ordered into scaffolds, without information about their relative orientation or chromosomal origin, because thoroughly annotated reference genomes and physical maps are still lacking for most taxonomic groups. In addition, many mammalian genomes have been sequenced from female individuals, to obtain equal coverage of autosomes and the X chromosome (Hughes and Rozen 2012), but also for technical difficulties relating to the assembly and the high amount of repetitive and ampliconic sequences on Y chromosomes (Willard 2003; Bachtrog 2013). This has hampered sequencing, assembly, identification, and application of Y-chromosomal markers (Greminger et al. 2010). As a consequence, complete Y chromosome sequences are only published for four mammalian species: Human, chimpanzee, rhesus macaque, and mouse (Skaletsky et al. 2003; Hughes et al. 2010, 2012; Soh et al. 2014). This list is complemented by large-scale analyses of Y-chromosomal sequences for dog, cat, marmoset, rat, bull, opossum, *Drosophila,* and medaka fish (Kondo et al. 2006; Carvalho et al. 2009; Li et al. 2013; Bellott et al. 2014).

In this study, we utilize a previously published polar bear reference assembly that is based on a male sequenced at high coverage (Li et al. 2011), and available short sequence reads from additional male and female polar bears (Miller et al. 2012). We identify Y-linked scaffolds by 1) searching for sequence similarity using known mammalian Y-linked gene sequences and 2) identifying scaffolds with sex-specific sequencing coverage characteristics indicative of Y linkage. The latter approach makes use of differences in the expected sequence coverage of male versus female sequence reads on autosomal, X-chromosomal, and Y-chromosomal reference scaffolds. We apply stringent quality filters to minimize false positives, that is, scaffolds wrongly identified as Y-linked. In addition, in vitro amplification of the longest candidate scaffolds confirmed the in silico findings. We demonstrate that genome scale Y-chromosomal sequences can be reliably identified from high-throughput sequencing data, also in organisms lacking a chromosome-based physical map of the genome.

## Materials and Methods

We used two different approaches to identify Y-chromosomal sequences in the recently published polar bear genome assembly from a male individual that was sequenced at 101-fold coverage (Li et al. 2011). This assembly has a size of 2.3 Gb and is arranged into 72,214 scaffolds with an N50 value of 15.9 Mb. Information regarding chromosomal locations and the relative orientation of the scaffolds is not available. Thus, it is unknown which of the scaffolds are of Y-chromosomal origin. In the following, we refer to this genome assembly as the “polar bear assembly,” and to the scaffolds of this assembly by their respective scaffold ID numbers.

### Similarity Search of Y-Linked Genes Lists Candidate Scaffolds

The first approach was to use 32 genes known to be Y-linked in other mammals as queries for a similarity search in the polar bear assembly. Exon sequences from human (*Homo sapiens*), mouse (*Mus musuculus*), chimpanzee (*Pan troglodytes*), and dog (*Canis lupus familiaris*) were downloaded from GenBank for these genes (supplementary table S1, Supplementary Material online). Similarity between exon sequences and the scaffolds of the polar bear assembly was identified using Basic Local Alignment Search Tool (BLAST), analyzing one exon at a time.

Scaffolds from the polar bear assembly were extracted from the list of BLAST hits according to the following criteria: 1) The scaffold with the lowest *E* value (expect value) for a particular exon relative to all other scaffolds in the list and 2) scaffolds with ≥95% sequence similarity compared with the scaffold with the lowest *E* value, with the additional constraint that the difference in alignment length of exon and scaffold (compared with the scaffold with the lowest *E* value) must not exceed 5%. We then obtained the exact position of each exon on its respective scaffold by realigning exon and scaffold using ClustalW. Only scaffolds with a sequence identity of ≥80% between scaffold and exon were kept (Table 1).

**Table 1:** Polar Bear Scaffolds Showing Similarity to 18 Mammalian Y-Linked Genes

Nr.	Scaffold (ID)	Scaffold Size (kb)	Gene
1	*13*	26,707	*RPS4Y*
2	*20*	22,125	*EIF1AY, EIF2S3Y, USP9Y, ZFY*
3	*46*	15,941	*TBL1Y*
4	*53*	14,458	*SLY*
5	*104*	6,801	*NLGN4Y, PRKY, TBL1Y*
6	*105*	6,717	*RBMY1A1*
7	*115*	5,608	*AMELY*
8	*134*	4,673	*UBA1Y, UTY*
9	*184*	2,589	*DDX3Y, USP9Y*
10	*186*	2,578	*PCDH11Y*
11	*253*	821	*RPS4Y, RPS4Y2*
12	*297 *^a^	391	*EIF1AY, KDM5D*
13	*301*	351	*KDM5D*
14	*309 *^a^	317	*DDX3Y, USP9Y, UTY,*
15	*318 *^a^ (*3836*)^b^	237	*EIF2S3Y, KDM5D, USP9Y, ZFY*^b^
16	*369 *^a^	104	*RBMY1A1*
17	*389 *^a^	77	*AMELY*
18	*403 *^a^	63	*UBA1Y*
19	*579 *^a^	21	*SRY*
20	*605 *^a^	19	*UBA1Y*
21	*646 *^a^	15	*EIF2S3Y*
22	*4889*	0.9	*UBA1Y*
23	*6612*	0.7	*AMELY*

^a^Scaffolds have an AD-ratio indicative of Y-linkage and were validated in vitro to be male-specific.

^b^The entire length of scaffold *3836* (1 kb; with similarity to *ZFY*) is included within scaffold *318* with 100% identity.

In addition, in vitro validated male-specific polar bear sequences from five known Y-linked genes (exons and introns, *AMELY*, *KDM5D* [*SMCY*], *SRY*, *UBA1Y*, *ZFY*; supplementary table S1, Supplementary Material online) (Nakagome et al. 2008; Pagès et al. 2008, 2009) were downloaded and used as a query against the polar bear assembly using BLAT, with default parameters. Polar bear sequences from the two X-linked genes *ZFX* and *AMELX* (Pagès et al. 2009) were used to differentiate between Y and X gametologs, that is, homologous gene copies on the X and the Y chromosomes (supplementary table S1, Supplementary Material online).

### Average Depth Ratio for Identification of Y-Linked, X-Linked, and Autosomal Scaffolds

In a second approach to identify Y-linked scaffolds, we utilized previously published short sequence reads from whole-genome sequence data of one female (SRX155950/PB06) and one male (SRX155954/PB10) polar bear. The two polar bear individuals had been sequenced at similar sequence depth (∼12×) on an Illumina HiSeq 2000 platform, generating paired-end reads (101 bp) with an insert size of about 400 bp (Miller et al. 2012). The AD ratio approach is based on differences in the relative numbers of X and Y chromosomes between females (2-0) and males (1-1), whereas both sexes carry two copies of each autosome. As unique Y-chromosomal sequences are not present in a female genome, reads obtained from genome sequencing of female and male individuals should map with characteristic sex-specific patterns to scaffolds from the Y chromosome, the X chromosome, and the autosomes. The expected differences in sequencing coverage were utilized primarily to identify Y-chromosomal scaffolds in the polar bear assembly, but our approach also allowed the assignation of anonymous scaffolds from the polar bear assembly as autosomal or X-linked.

Short read sequences were evaluated for residual adapter sequences and low-quality bases were clipped off the read-ends using FastQC v 0.10.0 (Andrews 2010) and sickle (Joshi and Fass 2011). BWA (Li and Durbin 2009) was used for the reference-guided mapping of the cleaned reads against the polar bear assembly. Using Samtools (Li et al. 2009), we merged read data from separate sequencing runs of the same sample into one single BAM file per individual. Picard (http://picard.sourceforge.net/, last accessed February 9, 2015) was used to mark duplicated reads, and realignment of reads was performed in GATK v2.3 (McKenna et al. 2010).

After mapping, the mpileup module of samtools was used to calculate the read depth at each position on a given scaffold for the male and the female genomes. Scaffolds without mapped reads or with low mapping quality (*n* = 614), and scaffolds that were shorter than 1 kb (*n* = 68,017; ∼15 Mb) were disregarded and not considered in the downstream analyses. For the remaining scaffolds (≥1 kb, *n* = 3,583), the average read depth was calculated: We determined the sum of the depth values at ambiguity-free scaffold positions (no “N”) with≤50 reads per position, and divided this by the number of ambiguity free scaffold positions.

Finally, the AD-ratio of each scaffold was calculated by dividing the average read depth in the female individual by the average read depth in the male individual (1). A normalization factor adjusted the number of female and male reads to each other (2): To this end, we divided the total number of reads (quality ≥ 20) in the female BAM file by that of the male BAM file.

For each given scaffold, average sequencing depth for the female and male genome was calculated using the following formulas:

(1) AD-ratio=average-depthfemale/ (average-depthmale×norm)

(2) norm=total number of readsfemale/ total number of readsmale

The normalization factor is used to enable comparison of read depth of individual scaffolds among individuals, despite possible differences in genome-wide sequencing coverage between them. Using this normalization factor, the male and female genomes are standardized to the same genome-wide average coverage. The AD-ratio is zero for perfectly mapped Y chromosome scaffolds, one for autosomal and two for X-linked scaffolds. For graphical representation, we combined scaffolds with different AD-ratios into bins of size 0.02.

### In Vitro Validation of Putative Y-Linked Scaffolds in Different Bear Species

To verify the male-specificity of scaffolds identified by the in silico analysis, we PCR-amplified fragments from 20 Y-scaffolds (table 2) in at least one male and one female individual of each of three closely related ursine bears: Polar bear, brown bear, and American black bear (*U. americanus*). In addition, amplification of fragments from two X-linked and two autosomal scaffolds as identified by the AD-ratio approach was made in both male and female bears to verify their non-Y-chromosomal origin. Before amplification, newly designed primers (supplementary table S7, Supplementary Material online) were tested in silico for unique binding by aligning the forward and reverse sequences against the scaffolds of the polar bear assembly using BLASTn. Scaffolds were defined as being Y chromosome specific when one clear amplification product was detected in males, but no amplicons or only low-intensity bands of different sizes were observed in females. In vitro experiments included touchdown PCRs (see supplementary material, Supplementary Material online) and agarose gel-electrophoresis to verify the expected size of the amplicons. Each PCR setup contained a no-template control.

**Table 2:** Y-Chromosomal Scaffolds ≥ 10 kb Identified by the AD-Ratio

Nr.	Scaffold ID	Size (kb)	AD-Ratio	Similarity to Y-Linked Gene
1	*297*	391	0.284	*EIF1AY, KDM5D*
2	*309*	317	0	*DDX3Y, USP9Y, UTY*
3	*318*	237	0.285	*EIF2S3Y, KDM5D, USP9Y, ZFY*
4	*322*	217	0.252	*—*
5	*369*	104	0.18	*RBMY1A1*
6	*389*	77	0.16	*AMELY*
7	*393*	70	0.12	*—*
8	*403*	63	0	*UBA1Y*
9	*420*	54	0	*—*
10	*519*	31	0.198	*—*
11	*579*	21	0.075	*SRY*
12	*596*	20	0	*—*
13	*605*	19	0	*UBA1Y*
14	*613*	18	0.158	*—*
15	*632*	16	0.205	*—*
16	*646*	15	0	*EIF2S3Y*
17	*657*	14	0	*—*
18	*771*	10	0.135	*—*
19	*795*	10	0	*—*
20	*813*	10	0	*—*

Note.—The male-specificity of all scaffolds listed here has been validated in vitro. Additional scaffolds (<10 kb) are shown in supplementary table S2, Supplementary Material online.

### Repetitive Element Estimation in the Polar Bear Genome and on Y-Linked Scaffolds

The amount of transposable elements (TE) on 14 of the larger validated scaffolds (scaffold IDs: *297*, *309*, *318*, *322*, *369*, *393*, *389*, *403*, *420*, *519*, *579*, *605*, *646*, and *657*; 1.6 Mb) was identified using RepeatMasker (http://www.repeatmasker.org, last accessed February 9, 2015) using the carnivore library (Smit et al. 1996). RepeatMasker with the carnivore library was also used to identify microsatellites with a minimum of 15 repeat units (supplementary table S6, Supplementary Material online).

### Analysis of X–Y Gene Conversion in Bears

The partial *ZFY* and *ZFX* exon sequences of all ursid species from Pagès et al. (2009) were downloaded from GenBank and aligned with homologous sequences from other mammals (397 bp) in Geneious 8.0.3 (Biomatters, Auckland, New Zealand). Based on the model suggested by jModeltest2 (Darriba et al. 2012), HKY+4 G, phylogenetic trees were constructed in Geneious, and a statistical parsimony network was generated in TCS (Clement et al. 2000).

### Calculation of Polar Bear Patrilineal Phylogeny

Five Y-linked scaffolds (IDs: *309*, *322*, *389*, *393*, and *403*) with a combined size of 743 kb were used to reconstruct the phylogenetic relationship of 12 polar bear individuals sampled in Svalbard (Norway) and Alaska. The Y-linked sequences were used to estimate the divergence time of the lineages within polar bears, using one American black and one brown bear as outgroup. Short reads of all 14 bear individuals (Miller et al. 2012) were retrieved from databases (supplementary table S8, Supplementary Material online) and mapped to the polar bear assembly as described above. The individuals have been labeled according to their respective description in the short read archive (supplementary table S8, Supplementary Material online). The five respective scaffolds together with previously mapped short reads were extracted using Samtools and loaded into Geneious 8.0.3 (Biomatters). Geneious was then used to create a consensus sequence for each individual, to align those consensus sequences, and to remove alignment columns containing ambiguous sites and gaps, respectively. Additionally, the alignments were manually inspected to find and remove columns where only one individual contained multiple differentiating sites adjacent to each other. This strict filtering reduced the size of the alignments by approximately 30% (see below).

A NeighborNet network was calculated in SplitsTree 4.12.6 (Huson and Bryant 2006) based on a 511-kb-long alignment of the concatenated Y-sequences of 12 polar bears. BEAST 2.1.3 (Bouckaert et al. 2014) was used to estimate divergence times among polar bears, using a strict clock model, a Yule tree model, and a uniform prior of 343-479 ka, based on the relatively young population divergence between brown and polar bears (Liu et al. 2014). An additional calibration scenario employed a fixed mutation rate obtained from human Y chromosomes: 0.76 × 10^−^^9^/site/year (Fu et al. 2014). We used the GTR+I substitution model as indicated by the Bayesian Information Criterion in jModeltest 2.1.1. Convergence was checked in Tracer (ESS > 200). The concatenated alignment comprising 506 kb included 12 polar bears, one brown bear, and one black bear. This alignment was thus slightly shorter than the polar bear alignment, due to ambiguous sites and gaps introduced by the inclusion of additional individuals/taxa.

## Results

We identified a total of 1.9 Mb of Y-chromosomal sequence data in the polar bear assembly, located on 112 different scaffolds. The scaffolds were identified by applying two different approaches: 1) The search for similarity of known Y-linked genes, and 2) comparison of the AD-ratio of reads from male and female genomes.

### The Similarity Search Identified 23 Putative Y-Chromosomal Scaffolds

**Fig. 1.—:**
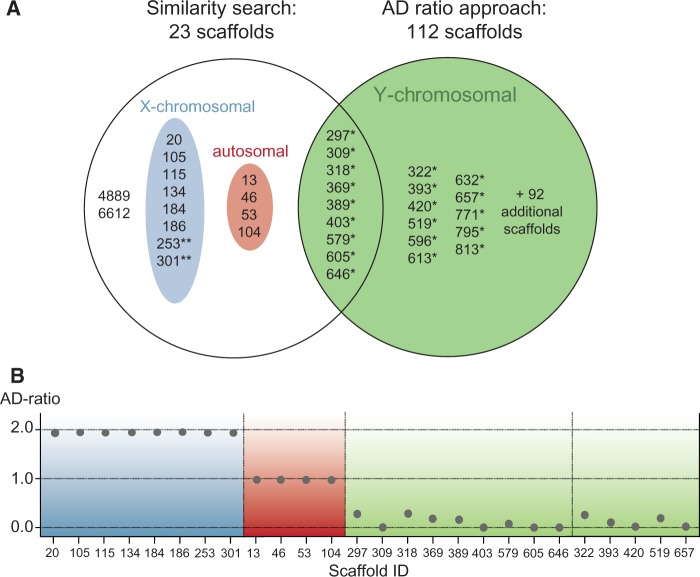
Identified scaffolds in the polar bear assembly. (*A*) Scaffolds identified by the similarity search, the AD-ratio, and by both approaches (overlap). Scaffolds ≥10 kb are shown by their ID numbers. Details for 92 additionally Y-linked scaffolds (<10 kb, combined length: ∼170 kb) are listed in supplementary table S2, Supplementary Material online. Some scaffolds identified by the similarity search showed AD-ratio characteristic of autosomal linkage (red) or X-linkage (blue). Scaffolds with an asterisk (*) have been verified in vitro to be male-specific. Two asterisks indicate scaffolds that show PCR amplification in both sexes. No reads mapped with sufficient mapping quality to scaffold *4889*, so its AD-ratio could not be calculated, and scaffold *6612* was shorter than 1 kb. (*B*) AD-ratios of X-linked (blue), autosomal (red), and Y-linked (green) scaffolds.

The first approach identified scaffolds in the polar bear assembly that showed similarity to known Y-linked gene sequences from four different mammals (human, mouse, chimpanzee, and dog). Exons from 18 of 32 Y-linked candidate genes that were blasted against the polar bear assembly identified polar bear scaffold sequences above a threshold of 80% identity (table 1 and supplementary table S1, Supplementary Material online). The hits were distributed across 23 scaffolds, ranging from 0.7 to 26,707 kb in size (table 1 and fig. 1). The full sequence length of scaffold *3836* (1,069 bp) had an identical sequence stretch on scaffold *318* (237 kb), with no nucleotide mismatches. Thus, we do not report scaffold *3836* as a distinct scaffold, although it is a separate entry in the current polar bear assembly.

Six sequences of five Y-linked genes from polar bear Y chromosomes (Nakagome et al. 2008; Pagès et al. 2008, 2009) aligned to the polar bear assembly with 98.9–100% identity. A 227-bp fragment from *ZFY* and a 49-bp fragment from *KDM5D* (*SMCY*) were uncharacterized (“N”) in the polar bear assembly.

We found that ten query genes had similarity to two or more different scaffolds in the polar bear assembly, thereby creating combinations of scaffolds that contain stretches of homologous sequence (supplementary table S4, Supplementary Material online). These scaffold combinations consisted of one in vitro validated Y-scaffold and one (or two) scaffold(s) with an AD-ratio expected for X-chromosomal linkage, indicating sequential homology between the Y-chromosomal and other scaffolds. For instance, the *ZFY* exon sequences mapped to both scaffold *318* and scaffold *20* with similar identity (99.2% vs. 99.5%). The gametologous polar bear *ZFX* sequence also mapped to both these scaffolds, at the same location as *ZFY.* However, when using less conserved intronic sequences from polar bears (supplementary table S1, Supplementary Material online) in a BLAT search against the polar bear assembly, scaffolds *318* (containing *ZFY*, Y-linked) and scaffold *20* (containing *ZFX*, X-linked) were clearly diagnosable.

Phylogenetic analyses of *ZFX*/*ZFY* sequences in mammals showed that the X- and Y-linked copies of giant panda (*Ailuropoda melanoleuca*) form a cluster, and that all ursine and tremarctine *ZFX*/*ZFY* sequences form a second cluster of closely related sequences (supplementary fig. S1, Supplementary Material online). Ursid sequences thus clustered together, regardless of their X- or Y-chromosomal origin. Other mammals clustered outside the ursid variation.

Based on the similarity of known Y-linked candidate gene sequences from different mammals, the similarity search provided us with a list of 23 scaffolds that might potentially be located on the polar bear Y chromosome. However, 12 of these scaffolds were identified to be autosomal or X-linked, due to their respective AD-ratios (see below, fig. 1 and supplementary table S4, Supplementary Material online).

### The Average-Depth Ratio Identified 112 Y-Chromosomal Scaffolds

**Fig. 2.—:**
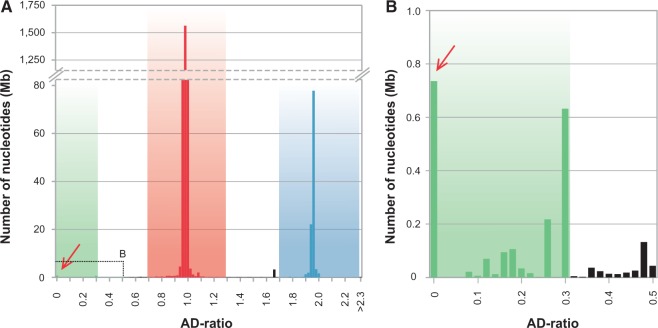
AD-ratio histogram of polar bear scaffolds. (*A*) Distribution of AD-ratios of scaffolds ≥1 kb, and their combined size shown in bins of width 0.02. Autosomal scaffolds cluster around an AD-ratio of 1 (red), X-linked scaffolds around 2 (blue). The stippled box highlights the region shown enlarged in (*B*). Enlargement of the box in (*A*). Scaffolds below the threshold of 0.3 are identified as Y-linked (green). Scaffolds unassigned to chromosomal classes are shown in black.

Most scaffolds had an AD-ratio of either approximately 1 or approximately 2, indicative of autosomal and X-chromosomal scaffolds, respectively (fig. 2 and supplementary fig. S2, Supplementary Material online). The combined sequence length of all putative autosomal scaffolds ≥ 1 kb (0.7 < AD-ratio < 1.3; *n* = 2,618) was approximately 2.18 Gb, and putative X-linked scaffolds (1.7 < AD-ratio < 2.3; *n* = 214) amounted to approximately 109 Mb. At an AD-ratio of zero, which is the expected AD-ratio for Y-linked scaffolds, we detected 90 scaffolds with a combined sequence length of 686 kb (fig. 2 and supplementary fig. S2, Supplementary Material online, table 2 and supplementary table S2, Supplementary Material online). An additional 22 scaffolds with a combined sequence length of 1.21 Mb showed AD-ratios ≤ 0.3, of which 11 were amplified in vitro, all showing male-specific amplification (table 2). Thus, applying a relaxed AD-ratio cutoff of ≤ 0.3, thereby allowing for a certain proportion of wrongly mapped reads, identified 112 Y-linked scaffolds, comprising 1.9 Mb of Y-chromosomal sequence.

Nine scaffolds totaling 1.24 Mb were identified by both approaches (fig. 1 and supplementary table S3, Supplementary Material online). Among the scaffolds obtained exclusively from the similarity search, one had an AD-ratio of exactly zero, but it was less than 1 kb (*scaffold ID 6612*; 794 bp). Four putative Y-chromosomal scaffolds from the similarity search had an AD-ratio of approximately 1, indicating autosomal origin. Eight scaffolds from the similarity search had an AD-ratio of approximately 2, indicating X-linked origin. For one scaffold identified by the similarity search, neither male nor female reads mapped with sufficient quality (scaffold ID *4889*), precluding any linkage classification.

### In Vitro Amplification Validates All Tested Y-Linked Scaffolds as Being Male-Specific

The male-specificity of the longest putative Y-linked scaffolds (*n* = 20) was additionally evaluated in vitro by PCR amplification (table 2). At least one fragment of 635–800 bp sequence length of each of the scaffolds was PCR-amplified using male DNA samples along with female DNA controls of each brown, polar and black bears. All 20 scaffolds showed male-specific PCR amplification, defined as the occurrence of a clear amplicon of a distinct size in males but not in females. In female DNA samples, the Y-chromosomal fragments could either not be amplified (scaffold IDs *309, 318, 322, 369, 389, 393, 420, 579, 596, 605, 613, 632,* and *813*) or the observed amplicons were smaller, with multiple low-intensity (unspecific) bands/smears on agarose gels (scaffold IDs *297, 403, 519, 646, 657, 771,* and *795*). For comparison, we validated two fragments with putative autosomal (scaffold IDs *236* and *267*) or X-linkage (scaffold IDs *301* and *253*), based on results from the AD-ratio approach. Markers on these putatively non Y-linked scaffolds could be PCR-amplified in both male and female DNA samples, and showed clear amplicons of the same sizes in both sexes.

### High Abundance of Repetitive Elements on the Y-Linked Scaffolds

**Fig. 3.—:**
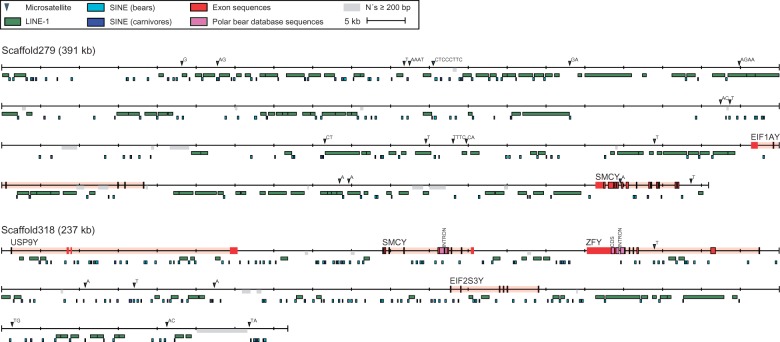
Annotations of Y-linked scaffolds *297* and *318.* Exons homologous to mouse and human are shown in red. Previously published Y-linked polar bear sequences are shown in pink. The repeat unit of each microsatellite is indicated and regions with greater than 200 bp of consecutive “N” are highlighted in gray. Due to the high abundance, only placental mammalian non-LTR retrotransposons ≥500 bp (LINEs) and ≥100 bp (SINEs) were plotted. The maps of additional scaffolds are shown in supplementary figure S3, Supplementary Material online.

Overall, TEs covered 54.38% of the total length of the 14 Y-scaffolds used in this analysis (supplementary tables S5 and S6, Supplementary Material online, fig. 3 and supplementary fig. S3, Supplementary Material online). The majority of the TE sequences represents placental mammalian LINE-1 (38%) or the carnivore CAN-SINEs (7.8%). The average LINE-1 coverage of the polar bear genome is 16.93%, thus LINE-1 covered nearly twice as much sequence on the Y chromosome scaffolds compared with the entire genome. In addition, one full-length LINE-1 copy, the L1-1_AMe, with a length = 6,021 bp was found on scaffold *297* (fig. 3). The full-length L1-1_AMe is likely to have been recently active, due to the presence of only two stop codons in the endonuclease/reverse transcriptase encoding ORF2. The abundance of repetitive regions along the Y chromosome, and the positions of homologous regions to candidate gene sequences are shown in figure 3, exemplary for two long Y-chromosomal scaffolds (scaffold IDs *297* and *318*). Corresponding maps for 12 additional Y-scaffolds are provided in supplementary figure S3, Supplementary Material online. We found a higher abundance of LINE-1 elements and a lower abundance of older LINE-2 and LINE-3 elements on the Y chromosome compared with the whole genome. Moreover, a higher abundance of carnivore-specific SINEs as compared with ancestral Mammalian Interspersed Repeats (MIR) was detected. We identified a similar amount of long terminal repeats (LTRs)/endogenous retroviruses (ERVs) on the Y chromosome and the whole genome, whereas less DNA transposons were identified on the Y chromosome compared with the whole genome. We identified 115 microsatellites with at least 15 repeat units, many of which are likely to show intraspecific polymorphism and are thus useful for population genetic studies, covering 0.3% of the combined length of all Y-scaffolds (supplementary table S6, Supplementary Material online).

### Phylogenetic Analyses Identify Two Distinct Male Polar Bear Lineages

**Fig. 4.—:**
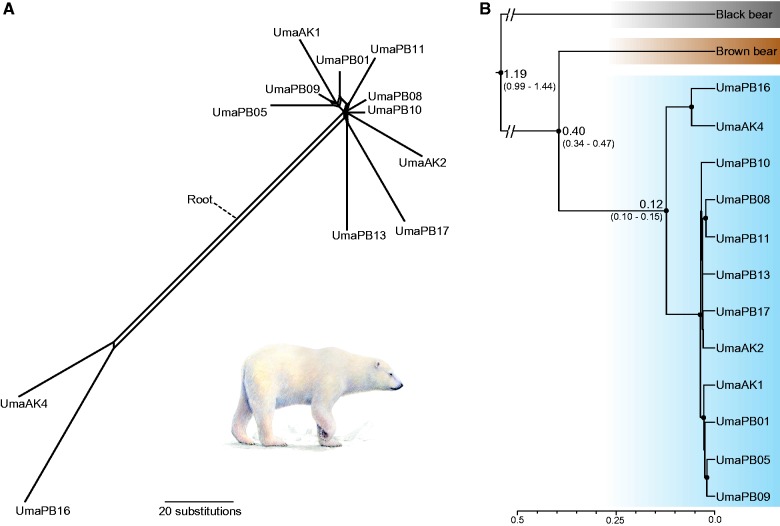
Phylogenomic analysis of approximately 0.5-Mb Y-chromosomal sequence from 12 polar bears. Geographic origins of the polar bear individuals are denoted by AK (Alaska) and PB (Svalbard). (*A*) NeighborNet analysis. (*B*) Time-calibrated Bayesian coalescent-based phylogeny from BEAST. Numbers at nodes indicate the median of the divergence time in million years ago, with 95% highest posterior density in brackets. Dots at nodes indicate posterior probability greater than 0.99. The scale axis is in units of million years ago. Note that approximately 80% older dates were retrieved from an alternative calibration scenario (see text).

Phylogenetic analysis of 511 kb Y chromosome sequence in 12 polar bears identified two highly divergent paternal lineages (fig. 4*A*), with two individuals (AK4 and PB16) being clearly separated from the remaining ten polar bears. This separation does not correspond to geography, as both major lineages occur in Alaska and Svalbard (Norway). Some individuals have a considerable number of unique substitutions (e.g., PB16: 55 substitutions) relative to 101 substitutions separating the two lineages. Our Bayesian analysis yielded a phylogenetic tree with high posterior support for all major nodes (fig. 4*B*), showing two distinct patrilineal clades within polar bears. Based on the demographic split of brown and polar bears at 343–479 ka (Liu et al. 2014), we obtained a median divergence time estimate for the split of these two clades at 0.12 Ma (95% highest posterior density (HPD): 0.10–0.15). The split between brown and polar bears was estimated at 0.40 Ma (0.34–0.47), and the divergence of the black bear at 1.190 Ma (0.99–1.44). Using a fixed mutation rate as an alternative calibration scenario, older divergence time estimates were obtained: The split within polar bears was estimated at 0.22 Ma (0.19–0.25), the split between brown and polar bears at 0.70 Ma (0.65–0.76), and the divergence of the black bear 2.13 Ma (2.03–2.23).

## Discussion

The Y chromosome is poorly characterized in most mammals, including the carnivoran bear family. We used the polar bear reference assembly to identify a large amount of Y-linked sequence, totaling 1.9 Mb distributed across 112 Y-linked scaffolds. We did so by applying a similarity search with mammalian Y-linked genes and by analyzing differences in sequencing coverage (AD-ratio) on Y-chromosomal, autosomal, and X-linked scaffolds. We verfied these in silico results in vitro by validating male-specificity by PCR amplification for 20 of the largest scaffolds, corresponding to 1.7 Mb of Y-chromosomal sequence (fig. 1 and tables 1 and 2).

### The AD-Ratio Approach Can Reliably Identify Scaffolds from Autosomes, the X and the Y Chromosomes

Using the AD-ratio approach, the majority of scaffolds in the polar bear assembly could be assigned to one of three chromosomal classes. We identified 2.18 Gb of autosomal sequence, which is close to the total size of the polar bear assembly of 2.3 Gb (fig. 2). The X-linked scaffolds amounted to 109 Mb, which approximates two-thirds the size of the human X chromosome (Ross et al. 2005). This scaffold class included the 12 scaffolds that were previously identified as being X-linked in bears (Cahill et al. 2013). The amount of identified autosomal and X-linked sequences thus fit the expectations for a typical mammalian genome of approximately 2–3 Gb (Rogers and Gibbs 2014). Only approximately 0.2% (∼5.3 Mb) of the polar bear assembly remained unassigned, because AD-ratios for these scaffolds were beyond our thresholds for autosomal, X-chromosomal, and Y-chromosomal sequences. This illustrates the reliability of the AD-ratio approach, and its suitability to screen a genome assembly for the three chromosome classes. The 1.9 Mb identified to be Y-linked is a considerable amount of Y-chromosomal sequence, given the lack of Y-linked genomic sequences for many mammals, the generally small size of the mammalian Y chromosome, and its highly repetitive nature that impedes assembly. We likely underestimate the total amount of Y-linked sequences (see below), and 1.9 Mb represents only a small fraction of the entire polar bear Y chromosome. The size of Y chromosomes differs considerably among mammals and even among carnivores, but Y chromosomes are typically longer than 20 Mb (e.g., dog: 20 Mb, cat: 45 Mb; Li et al. 2013). Although the size of the polar bear Y chromosome has not yet been determined, it appears to be about half the physical size of the X chromosome in metaphase spreads (O’Brien et al. 2006).

The similarity search identified 23 scaffolds as being Y-linked; however, later inspection indicated that only nine of these had an AD ratio indicative of Y-linkage (fig. 1 and table 1 and supplementary table S3, Supplementary Material online). The AD-ratio approach yielded 112 Y-linked scaffolds and thus proved to be more efficient than the similarity search in terms of scaffold numbers. However, the nine scaffolds identified by both approaches total 1.24 Mb, which is more than 60% of the entire Y-linked sequence data. Although a similarity search is technically simple, successful, and easily applied, several drawbacks are associated with this approach.

We based our selection of query genes on their previous description as being Y-linked in other mammals, implicitly assuming the presence of these genes also on the polar bear Y chromosome. However, Y chromosomes can differ in their gene content across taxa, and lineage-specific sets of Y-linked genes exist (Murphy et al. 2006; Cortez et al. 2014). Indeed, we found that Y-linked genes that are absent in carnivores, for example, *NLGN4Y* (Cortez et al. 2014), were also absent in polar bears. In contrast, genes that are widespread throughout placental mammals, and occur in other carnivores (dog and cat; e.g., *ZFY*, *UTY*, *EIF1AY*) (Cortez et al. 2014), are those genes that are actually found on Y-linked scaffolds in the polar bear assembly (fig. 3 and supplementary fig. S3, Supplementary Material online). Currently limited knowledge of gene contents on the Y chromosomes of different mammalian lineages is therefore still restricting the efficiency of similarity-based approaches for the identification of Y-chromosomal scaffolds.

In several cases, the similarity search produced hits to more than one scaffold (supplementary table S4, Supplementary Material online). For example, a search with *ZFY* sequences yielded similarity to scaffold *318* (containing *ZFY*) and scaffold *20* (containing *ZFX*). The scaffolds in such groups all had AD-ratios characteristic of either Y- or X-linkage (supplementary table S4, Supplementary Material online). Most Y-linked genes on these scaffolds are classified as X-degenerate in humans (supplementary table S1, Supplementary Material online). These genes are relics of the ancient autosomes from which the mammalian X and Y chromosomes evolved, and are thus expected to show homology between the X and Y chromosomes (Skaletsky et al. 2003). In contrast, *RBMY* (scaffold IDs *369* and *105*; supplementary table S4, Supplementary Material online) is classified as ampliconic in humans, and such genes normally lack X-linked counterparts. *RBMY*, however, is one of the two ampliconic genes with an X-linked homolog in humans (*RBMX*), explaining its detection on an X-linked scaffold. These findings illustrate the high degree of sequence similarity between some sex chromosome gametologs (homologous genes on the two sex chromosomes), and the common evolutionary history of Y and X chromosomes, deriving from an ancestral pair of autosomes. Moreover, four scaffolds with sequence similarity to Y-linked genes, but an AD-ratio indicative of autosomal (or pseudoautosomal) origin, were identified (supplementary table S3, Supplementary Material online). Interestingly, one of these genes (*RPS4Y*, on scaffold: *13*) is in close proximity to the pseudoautosomal region (PAR) on the small arm of the human Y chromosome (Skaletsky et al. 2003). The location of the PAR is not known in polar bears, but genes in regions recombining with the X chromosome would hinder correct identification of Y-linked scaffolds by the AD-ratio approach.

The similarity search is further complicated by the high degree of similarity between some gametologous genes. This is exemplified by *ZFY*/*ZFX* genes, for which we were initially not able to differentiate between the respective Y- and X-scaffolds based on exon sequences. The more rapidly evolving intron sequences, however, allowed us to differentiate between Y- and X-linked scaffolds (supplementary fig. S1, Supplementary Material online). With a more stringent set of candidate genes, that is, carnivore-specific Y-linked genes, the reliability of the similarity search can be improved, and the search for intronic sequences would allow for a better differentiation between gametologs on the two sex chromosomes. A drawback of a similarity search based on Y-linked gene sequences from other taxa is that scaffolds consisting of exclusively intergenic sequence cannot be identified. This is an important limitation of similarity search approaches, because mammalian Y chromosomes are generally gene poor, with only 78 protein-coding genes in humans (Bachtrog 2013). Indeed, 103 of 112 Y-linked scaffolds were solely identified by their AD-ratio. Nevertheless, four of five Y-linked scaffolds with a size of greater than 100 kb were also identified by the similarity search (fig. 1 and table 2). Assemblies with fewer but larger scaffolds will thus be more amenable to accurate detection of Y-linkage by a similarity search approach.

### The Structure of the Y Chromosome Complicates Identification of Y-Linked Sequences

The heterochromatic, highly repetitive regions of a genome usually remain unassembled in whole-genome sequencing projects. Some Y chromosomes contain extended regions of largely uncharacterized heterochromatin, for example, human and *Drosophila* (Bachtrog 2013). Other Y chromosomes are largely euchromatic, for example, mouse and chimpanzee, but even the euchromatic regions are enriched for ampliconic sequences containing duplicated genes (Skaletsky et al. 2003; Hughes et al. 2010; Soh et al. 2014). Accurate sequence assembly is therefore inherently difficult for the Y chromosome, and sequence similarity to the X and possibly other chromosomes further complicates the identification of a distinct Y-linked sequence. Therefore, high-quality Y chromosome reference sequence assemblies are so far lacking from most mammalian genome sequencing projects.

The identification of Y-linked scaffolds has previously been achieved by in silico search for known Y-linked genes and massive in vitro PCR-based verification in *Drosophila* and *Anopheles* (Carvalho et al. 2000; Krzywinski et al. 2004). Moreover, Y-linked sequences can be retrieved by subtracting the scaffolds of the homogametic from the heterogametic assembly (Chen et al. 2014). Approaches based on some measure of the coverage depth of sequence reads on Y-linked scaffolds (Carvalho et al. 2003; Chen et al. 2012), for example, the “Y chromosome genome scan” (Carvalho and Clark 2013), or on the number of alignments in males and females, the “chromosome quotient” (Hall et al. 2013), have also been applied.

The occurrence of gene conversion, where a gene copy on one chromosome is overwritten by the information from the other chromosome, further complicates identification of chromosome-specific sequences. This process appears also to occur in the bear lineage (supplementary fig. S1, Supplementary Material online). Compared with the human and dog outgroups, the tremarctine and ursine *ZFY* and *ZFX* sequences cluster together, and not with human and dog *ZFY* and *ZFX*, respectively. Additionally, the *ZFY* and *ZFX* sequences from giant panda (*Ailuropoda melanoleuca*) are more closely related to each other than any gene copy is to those from ursine and tremarctine bears. A likely explanation for these observations is that gene conversion has occurred in the ancestral giant panda lineage as well as in the lineage leading to tremarctine and ursine bears. Considering the divergence times of ursid lineages, these two conversion events occurred in the Miocene, more than 12 and 6 Ma, respectively (Kutschera et al. 2014). The occurrence of gene conversion between sex chromosomes has been described in various mammalian lineages such as primates and felids (Slattery et al. 2000; Rosser et al. 2009; Trombetta et al. 2014), including *ZFX*/*ZFY.*

TEs on the sex chromosomes pose yet another challenge for accurate assembly and identification of chromosome-specific sequences. Mammalian genomes contain large amounts of TEs that propagate through different mechanisms. The human genome has over 44% of TEs (Lander et al. 2001), whereas the polar bear genome consists of 39.2% TEs (supplementary table S5, Supplementary Material online). Previous studies have shown that there is a preferential insertion of some TEs (primate-specific LINE1 and Alu elements) on the human and chimpanzee X and Y chromosomes (Kvikstad and Makova 2010). The same distribution is observed on polar bear Y chromosome scaffolds, as there is a high abundance of LINE-1 and the carnivore-specific Can-SINEs (Walters-Conte et al. 2011) compared with the autosomes (supplementary table S5, Supplementary Material online). The ancestral TEs, such as LINE-2, LINE-3 and MIR elements which were active before the split between marsupial and placental mammals (Smit and Riggs 1995), are found in very low numbers on the polar bear Y scaffolds. The ERV and DNA transposons seem to accumulate more evenly across the genome than LINE-1 and Can-SINEs, as there are only small differences between Y-chromosomal and autosomal scaffolds. The reason for the preferential accumulation on the sex chromosomes has been attributed to male and female germline TE integrations occurring before meiotic sex chromosome inactivation (Kvikstad and Makova 2010).

Due to the repetitive nature of the Y chromosome, assembly methods will likely produce numerous smaller scaffolds and collapse repetitive sequences into chimeric scaffolds that actually comprise multicopy sequences. Indeed, stretches of very high sequence coverage were found on many of the Y-linked scaffolds. Moreover, long and highly repetitive regions of the Y chromosome might be entirely missing from the assembly. TEs and X-transposed sequences on the Y chromosome likely cause a proportion of female reads from polar bear X chromosome and autosomes to be falsely mapped to Y-linked scaffolds, due to the high similarity among such regions. This produces AD-ratios greater than zero for these true Y-scaffolds. Finally, due to the paucity of information on bear sex chromosomes, we cannot exclude the possibility of recent stratum formation, with the existence of segments that have not yet attained a high level of divergence between the Y and X chromosomes. Yet, recent stratum formation or added genes from autosomes have not been reported for the well-studied Y chromosomes of two other carnivores, cat and dog (Cortez et al. 2014). A strict AD-ratio threshold of exactly zero is therefore likely to produce many false negatives. Our employed relaxed AD-ratio threshold of ≤0.3 yielded an additional 22 scaffolds, 11 of them tested and verified in vitro (table 2) to be of Y-chromosomal origin.

Assembly artifacts resulting from the repetitive nature of the Y chromosome imply that we likely underestimate the actual number and length of the identified Y-linked sequences. The AD-ratio approach should thus not be seen as an attempt to identify all Y-linked sequences in bears, nor to determine the size of the polar bears’ Y chromosome. Rather, the approach is an effective means to identify sequences that demonstrably have a high probability of being Y-linked and that can be used for evolutionary studies.

### Y-Chromosomal Sequences Provide a High Resolution Patrilineal Perspective on Polar Bear Evolutionary History

Our phylogenetic analyses of Y-linked scaffold sequences provide a patrilineal view on polar bear evolution that support a previously identified pattern of two distinct Y-chromosomal lineages in polar bears, PO1.1 and PO2 (Bidon et al 2014) (fig. 4). The large amount of analyzed sequence data provides high resolution of individual lineages, with many haplotype-specific substitutions. Our divergence time estimation places the split of these two polar bear clades around the Eemian interglacial period (0.12–0.13 Ma), implying that the two lineages separated long before the last glacial maximum (approximately 18–25 ka). The clear separation into two paternal lineages indicates an ancient population structuring in polar bears, possibly due to the separation into multiple refugia during glaciation cycles, similar to other arctic species (Flagstad and Røed 2003).

The divergence time was estimated using a recently published date on the population split between brown and polar bears (343–479 ka; Liu et al. 2014). This demographic split is expected to be younger than estimates based on the coalescence of allelic lineages, for example, the 338–934 ka estimated by Hailer et al. (2012). The lower effective population size of the Y chromosome implies that coalescence of Y-lineages occurs faster than that of autosomal lineages. Therefore, the Y-chromosomal gene tree might track the demographic splits of the species more closely.

It is noteworthy that our divergence estimate of the black bear patriline (0.99–1.44 Ma 95% HPD) is relatively young in this calibration scenario. The fossil record suggests a first occurrence of the black bear lineage at least 1.8 Ma (Kurtén and Anderson 1980). In principle, a Y-specific mutation rate would be a reasonable alternative calibration method. However, an independent mutation rate for the ursid Y chromosome has not yet been determined, and lineage-specific rates in mammals make the adoption of a Y-specific rate from another taxon unreliable. Applying a recent estimate for the mutation rate of the human Y chromosome, we obtained even older divergence time estimates for the patrilines of polar bears (0.19–0.25 Ma), of brown and polar bears (0.65–0.76 Ma), and of the black bear lineage (2.03–2.23 Ma). These dates are broadly consistent with other estimates of genomic divergence times for these splits (Hailer et al. 2012; Cahill et al. 2013; Cronin et al. 2014), and more in line with the fossil record of American black bears.

Short Y-linked sequences were recently used as markers for sex determination in bears (Bidon et al. 2013), phylogeographic analyses of brown and polar bear brother lineages (Bidon et al. 2014), and phylogenetic analyses of all eight bear species (Kutschera et al. 2014). Sequences on Y-chromosomal scaffolds have thus already proven to be a reliable resource for studying the evolutionary history of polar bears and other members of the ursid family.

## Conclusions

The analyses of Y-chromosomal scaffolds provided a high-resolution view on the patrilineal relationship within polar bears, identifying two highly distinct clades that separated during the middle Pleistocene. A preferential accumulation of younger TEs on the polar bear Y chromosome could be shown. As more and more genomes become available in the form of reference assemblies and short read archives, straightforward in silico strategies to identify sex-linked sequences from these data can now be applied in many species. Overall, the AD-ratio approach seems to be highly specific and preferable for a reliable identification of Y chromosome scaffolds. It can be used as long as a reference assembly of the heterogametic sex, and short reads of one male and one female are available.

## Supplementary Material

Supplementary tables S1–S8 and figures S1–S3 are available at *Genome Biology and Evolution* online (http://www.gbe.oxfordjournals.org/).

Supplementary Data
