# Supplementary material for: Genome-Wide Search Identifies 1.9 Mb from the Polar Bear Y Chromosome for Evolutionary Analyses
Source: Genome Biol Evol. 2015 May 27;7(7):2010–22. doi: 10.1093/gbe/evv103 (PMC4524476; doi:10.1093/gbe/evv103)
Supplement: Supplementary Data [file supp_7_7_2010__index.html]

Supplementary Data 

# Genome-Wide Search Identifies 1.9 Mb from the Polar Bear Y Chromosome for Evolutionary Analyses

## Supplementary Data

files

- Supplementary Data - pdf file
