# Supplementary material for: Genome-Wide Search Identifies 1.9 Mb from the Polar Bear Y Chromosome for Evolutionary Analyses
Source: Genome Biol Evol. 2015 May 27;7(7):2010–22. doi: 10.1093/gbe/evv103 (PMC4524476; doi:10.1093/gbe/evv103)
Supplement: Supplementary Data [file supp_evv103_BidonEtAl_suppl_revision_v2.pdf]

Supplementary information

Genome-wide search identifies 1.9 megabases from the polar bear Y chromosome for evolutionary analyses

Tobias Bidon, Nancy Schreck, Frank Hailer , Maria A. Nilsson and Axel Janke

Details on *in-vitro* experiments

Polymerase chain reaction (PCRs) were performed in 15 µl reaction volumes containing 2x Taq DNA Polymerase mix (VWR International GmbH, BDH Prolabo, Darmstadt, Germany), 0.17 µg/µl BSA (New England Biolabs, Ipswich, MA, USA), 0.27 µM each of forward and reverse primer, and 10-15 ng template DNA. The amplification protocol started with 95°C for 3 min followed by 14 cycles at 94°C for 30 s, a touchdown step for 25 s (see Table S7 for specific starting temperatures) and 72°C for 75 s. This was followed by 25 cycles of 94°C for 30 s, a specific annealing temperature for 25 s (Table S7) and 72°C for 75 s. Final elongation was conducted for 10 min at 72°C.

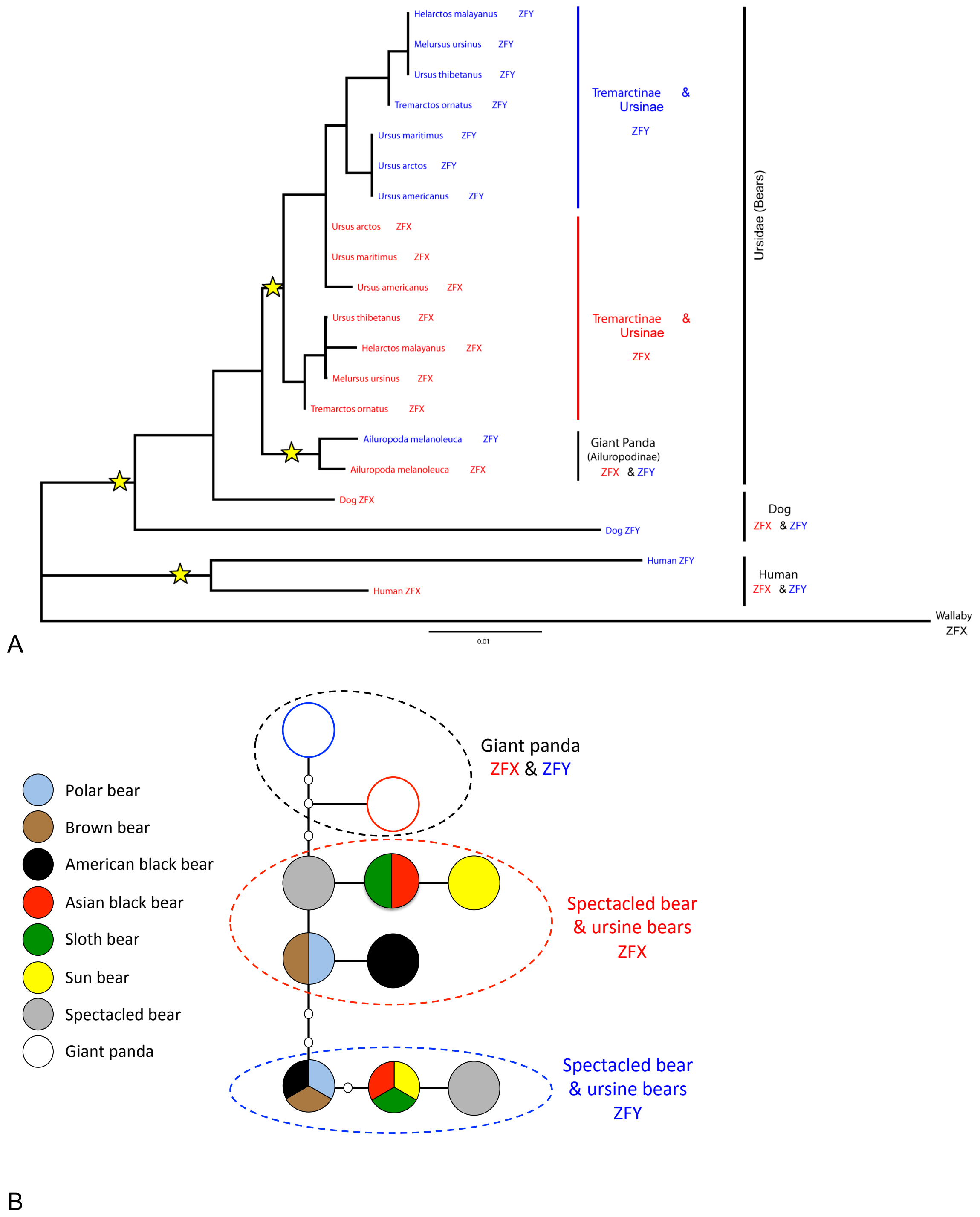

Figure S1: **Evidence for X/Y gene conversion events (yellow stars) in bears.**

A Rooted phylogeny of ~397 bp from *ZFY* and *ZFX* sequences in bears and other mammals. ***B*** Statistical haplotype network of bear *ZFY* and *ZFX* exon sequences (bear sequences from ***A***).

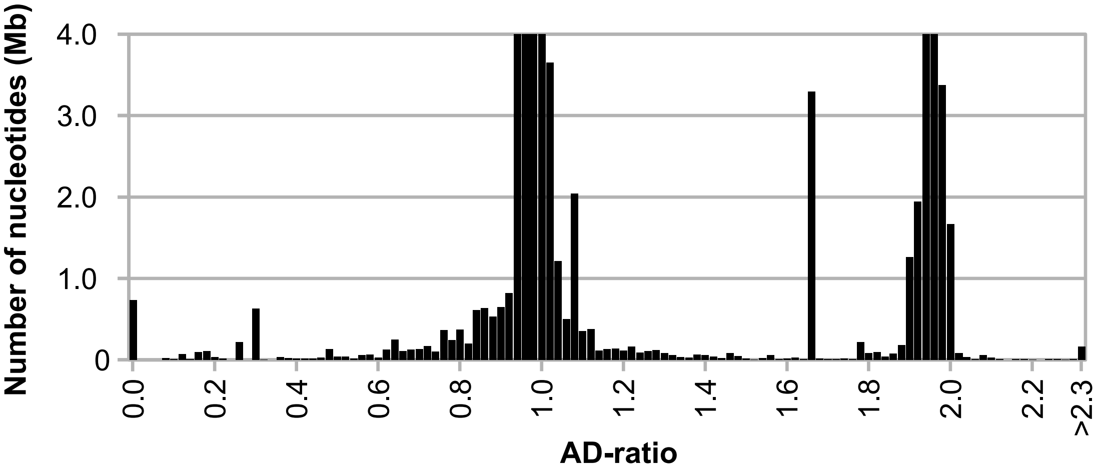

Figure S2: Average-depth (AD) ratio for scaffolds in the polar bear assembly (enlarged version of Fig. 2A).

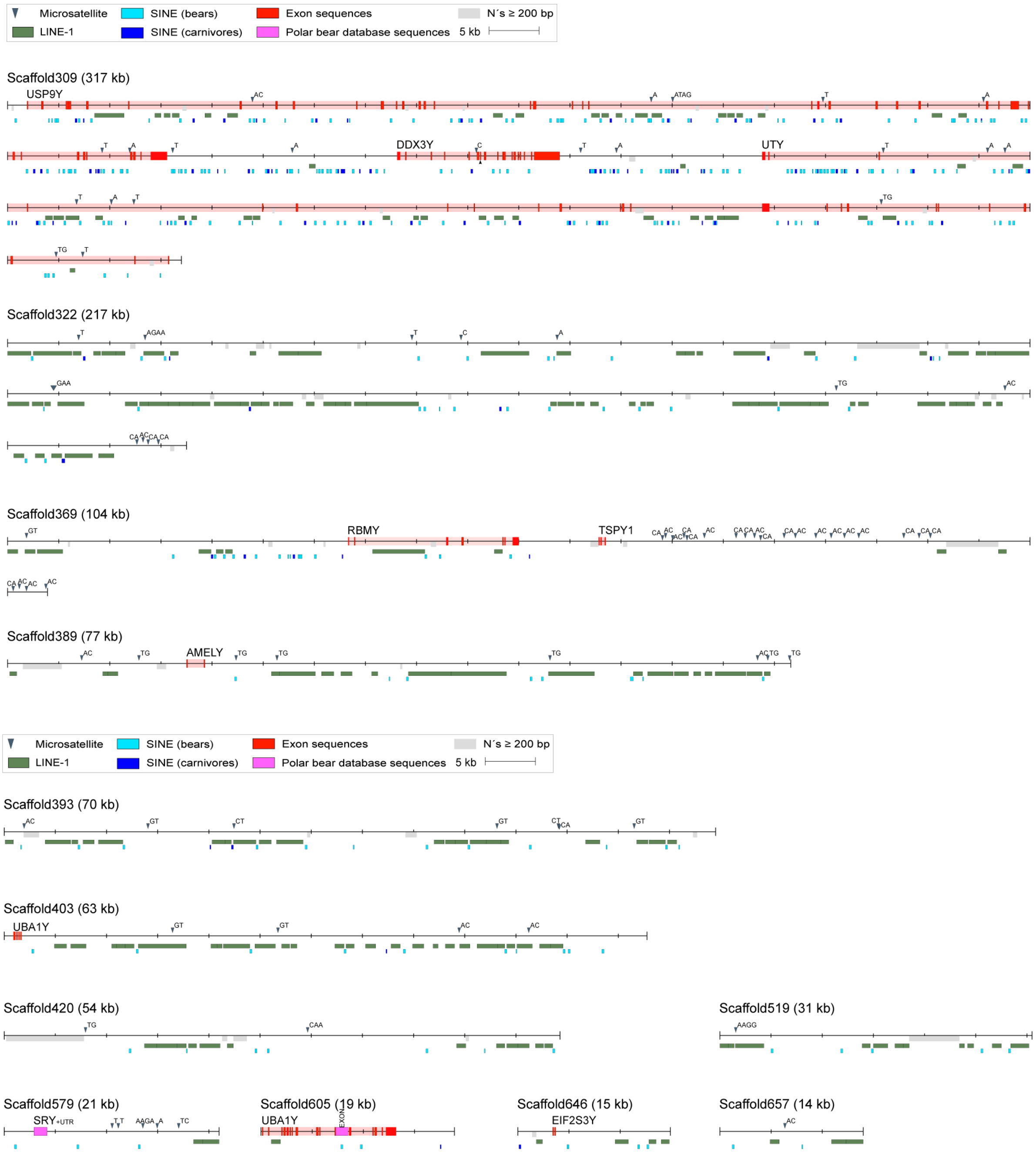

Figure S3: Annotations of Y-linked scaffolds.

Exons homologous to mouse and human are shown in red. Previously published Y-linked polar bear sequences are shown in pink. The repeat unit of each microsatellite is indicated and regions with >200 bp of consecutive "N" are highlighted in gray. Due to the high abundance, only placental mammalian non-LTR retrotransposons ≥500 bp (LINEs) and ≥100 bp (SINEs) were plotted. The maps of additional scaffolds are shown in **Figure 3**.

Table S1: Gene sequences used in the similarity search. Previously published polar bear sequences are in bold. Asterisk indicates the 18 mammalian genes that were found to have identity ≥80% to scaffolds of the polar bear assembly (see Table 1).

No.	Gene	Sequence class in humans (according to Skaletsky et al. 2003)	Available sequences*	Fragment extracted	Species	GenBank accession number	Scaffold identified	Number of gene fragments recovered on respective scaffold
**Y-linked**								
1*	*AMELY*		Exon 5	exon	***Ursus maritimus***	AM941064.1	115	1 exon
	AMELY	X-degenerate	RefSeqGene	exon	*Homo sapiens*	NG_008011.1	389	2 exons
2	*BPY2*	Ampliconic	RefSeqGene	exon	*Homo sapiens*	NG_009862.1	-	-
3	*CDY1*	Ampliconic	RefSeqGene	exon	*Homo sapiens*	NG_011754.1	-	-
4*	*DDX3Y (DBY)*	X-degenerate	Intron 5 + partial CDS	exon	*Pan troglodytes*	JF293113.1	309	1 exon
	DDX3Y (DBY)		RefSeqGene	exon	*Homo sapiens*	NG_012831.1	184, 309	12 exons, 17 exons
	DDX3Y (DBY)		RefSeqGene	exon	*Mus musculus*	NM_012008.2	184, 309	9 exons, 15 exons
5*	*EIF1AY*	X-degenerate	mRNA	exon	*Homo sapiens*	NM_004681.2	20, 297	3 exons, 5 exons
	EIF1AY		Partial exon	exon	*Pan troglodytes*	AB176583.1	-	-
6*	*EIF2S3Y*		mRNA	exon	*Mus musculus*	NM_012011.1	20, 318, 646	8 exons, 6 exons, 2 exons
7	*HSFY1*	Ampliconic	RefSeqGene	exon	*Homo sapiens*	NG_012030.1	-	-
8*	*KDM5D (SMCY)*	X-degenerate	RefSeqGene	exon	*Homo sapiens*	NG_032920.1	297, 301, 318	12 exons, 9 exons, 8 exons
	KDM5D (SMCY)		mRNA	exon	*Mus musculus*	NM_011419.3	297, 301, 318	8 exons, 4 exons, 7 exons
	KDM5D (SMCY)		intron 4	complete (intron)	***Ursus maritimus***	AB261824.1	318	1 intron
9*	*NLGN4Y*	X-degenerate	RefSeqGene	exon	*Homo sapiens*	NG_028212.1	104	4 exons
10*	*PCDH11Y*	X-transposed	RefSeqGene	exon	*Homo sapiens*	NG_011652.1	186	3 exons
11*	*PRKY*	X-degenerate	mRNA	exon	*Homo sapiens*	Y15801.1	104	4 exons
12	*PRY*	Ampliconic	RefSeqGene	exon	*Homo sapiens*	NG_032916.1	-	-
13	*RBM31Y*		mRNA	exon	*Mus musculus*	NM_028970.1	-	-
14	*RBMY*		mRNA	exon	*Mus musculus*	NM_011253.2	-	-
15*	*RBMY1A1*	Ampliconic	RefSeqGene	exon	*Homo sapiens*	NG_012805.1	105, 369	1 exon, 6 exons
16*	*RPS4Y*		Partial CDS	exon	*Pan troglodytes*	AH012491.2	13, 253	1 exon, 6 exons
17*	*RPS4Y2*	X-degenerate	RefSeqGene	exon	*Homo sapiens*	NG_032924.1	253	5 exons
18*	*SLY*		mRNA	exon	*Mus musculus*	NM_201530.2	53	1 exon
19*	*SRY*		Exon 1	complete (exon +UTR)	***Ursus maritimus***	AM748305.1	579	1 exon + UTR
	SRY	X-degenerate	RefSeqGene	exon	*Homo sapiens*	NG_011751.1	-	-
	SRY		mRNA	exon	*Mus musculus*	NM_011564.1	-	-
20	*SSTY1*		mRNA	exon	*Mus musculus*	NM_009220.2	-	-
21	*SSTY2*		mRNA	exon	*Mus musculus*	NM_023546.3	-	-
22*	*TBL1Y*	X-degenerate	mRNA	exon	*Homo sapiens*	NM_033284.1	46, 104	1 exon, 12 exons
23	*TGIF2LY*	X-transposed	mRNA	exon	*Homo sapiens*	NM_139214.2	-	-
24	*TMSB4Y*	X-degenerate	mRNA	exon	*Homo sapiens*	NM_004202.2	-	-
25	*TSPY1*	Ampliconic	RefSeqGene	exon	*Homo sapiens*	NG_027958.1	-	-
26*	*UBA1Y*		mRNA	exon	*Mus musculus*	NM_011667.2	134, 403, 605, 4889	12 exons, 1 exon, 13 exons, 1 exon
	UBA1Y		Exon 18	complete (exon)	***Ursus maritimus***	AM748329.1	605	1 exon
27*	*USP9Y*	X-degenerate	RefSeqGene	exon	*Homo sapiens*	NG_008311.1	20, 184, 309, 318	1 exon, 34 exons, 38 exons, 1 exon
	USP9Y		mRNA	exon	*Mus musculus*	NM_148943.2	20, 184,	1 exon, 29 exons, 35

							309, 318	exons, 1 exon
28*	*UTY*		mRNA	exon	*Mus musculus*	NM_009484.2	134, 309	19 exons, 21 exons
29	*VCY*	Ampliconic	RefSeqGene	exon	*Homo sapiens*	NG_032915.1	-	-
30	*XKRY*	Ampliconic	RefSeqGene	exon	*Homo sapiens*	NG_032917.1	-	-
31*	*ZFY*		Final intron	complete (intron)	***Ursus maritimus***	AB261808.1	318	1 intron
	ZFY		partial gene	complete (partial gene)	***Ursus maritimus***	AM748297.1	20, 318	1 exon, 1 exon
	ZFY		Partial CDS	exon	*Canis lupus*	JX475923.1	20, 318	3 exons, 4 exons
	ZFY	X-degenerate	RefSeqGene	exon	*Homo sapiens*	NG_008113.1	20, 318	5 exons, 6 exons
32	*ZFY1*		mRNA	exon	*Mus musculus*	NM_009570.4	-	-
**X-linked**								
1	*AMELX*		Exon 5	exon	***Ursus maritimus***	AM941056.1	115, 6612	1 exon, 1 exon
2	*ZFX*		Final intron	complete (intron)	***Ursus maritimus***	AB261816.1	20	1 intron
3	*ZFX*		partial gene	complete (partial gene)	***Ursus maritimus***	AM941048.1	20, 318, 3838	1 exon, 1 exon, 1 exon

Table S2: Y-linked scaffolds <10 kb (n=92) identified by their AD-ratio.

Scaffold ID	Size (kb)	AD-ratio
882	9	0
949	6	0
951	6	0
955	6	0.085
983	5	0
1057	5	0
1166	4	0
1214	3	0
1229	3	0.3
1232	3	0
1235	3	0
1237	3	0
1251	3	0
1261	3	0
1273	3	0
1329	3	0
1346	3	0
1359	3	0
1484	3	0
1621	2	0
1629	2	0
1665	2	0
1687	2	0
1727	2	0
1741	2	0
1756	2	0
1760	2	0
1772	2	0
1850	2	0
1880	2	0
1885	2	0
1911	2	0
1934	2	0
1956	2	0
2053	2	0
2099	2	0
2138	2	0
2227	2	0
2256	2	0
2285	2	0
2428	2	0
2434	2	0
2433	2	0
2467	1	0
2483	1	0
2502	1	0

Scaffold ID	Size (kb)	AD-Ratio
2560	1	0
2698	1	0
2697	1	0
2720	1	0
2812	1	0
2853	1	0.136
2856	1	0
2882	1	0
2879	1	0
3005	1	0
3008	1	0.225
3026	1	0
3027	1	0
3043	1	0
3094	1	0
3105	1	0
3158	1	0
3258	1	0
3283	1	0.299
3349	1	0
3381	1	0.271
3481	1	0
3487	1	0
3578	1	0
3585	1	0
3598	1	0.196
3608	1	0
3649	1	0
3662	1	0.163
3691	1	0
3693	1	0
3723	1	0
3838	1	0
3849	1	0
3886	1	0
3889	1	0
3894	1	0
3958	1	0.184
3965	1	0
4014	1	0
4021	1	0.182
4107	1	0.172
4138	1	0
4147	1	0
4146	1	0
4157	1	0

Table S3: Scaffolds identified by the similarity search to be Y-linked, but with AD-ratios indicative of being autosomal or X-linked.

Scaffold ID	Size [Kbp]	AD-ratio	Inferred chromosomal location	Homology search
13	26,707	0.962	A	*RPS4Y*
20	22,125	1.942	X	*EIF1AY, EIF2S3Y, USP9Y, ZFY*
46	15,941	0.978	A	*TBL1Y*
53	14,458	0.973	A	*SLY*
104	6,801	0.969	A	*NLGN4Y, PRKY, TBL1Y*
105	6,717	1.944	X	*RBMY1A1*
115	5,608	1.934	X	*AMELY*
134	4,672	1.943	X	*UBA1Y, UTY*
184	2,589	1.949	X	*DDX3Y, USP9Y*
186	2,578	1.956	X	*PCDH11Y*
253	821	1.933	X	*RPS4Y, RPS4Y2*
301	351	1.928	X	*KDM5D*
4889 *	0.9	-	-	*UBA1Y*
6612 **	0.7	0	-	*AMELY*

* Reads mapped to scaffold *4889* with quality <20, so this AD-ratio could not be determined.

** Scaffold *6612* (749 bp) has an AD-ratio of zero but was filtered out due to its size <1kb.

Table S4: Scaffold combinations containing Y- and X-linked scaffolds

Details from the 10 query genes that were observed on ≥2 scaffolds are shown (see also Table S1).

Scaffold with AD-ratio indicative of Y-linkage and male-specific amplification in-vitro	Scaffold with putative X-linkage (AD-ratio in brackets)	Query Gene
297	*20* (1.94)	*EIF1AY*
297	*301* (1.94)	*KDM5D*
309	*134* (1.94)	*UTY*
309	*184* (1.95)	*DDX3Y*
309	*20* (1.94),<br>*184* (1.95)	*USP9Y*
318	*20* (1.94)	*EIF2S3Y, ZFY*
318	*301* (1.94)	*KDM5D*
318	*20* (1.94),<br>*184* (1.95)	*USP9Y*
369	*105* (1.94)	*RBMY1A1*
389	*115* (1.93)	*AMELY*
403	*134* (1.94)	*UBA1Y*
605	*134* (1.94)	*UBA1Y*
646	*20* (1.94)	*EIF2S3Y*
6612	*115* (1.93)	*AMELY*

Table S5: Percentage of transposable elements in 1.6 Mb Y-chromosomal scaffolds in comparison to the genome-wide average in polar and brown bears.

	Polar bear		Brown bear
	**Y-scaffolds**	**Genome**	**Genome**
**SINE**	8.23	8.18	8.15
**MIRs**	0.42	3.03	3.05
**CanSINE**	7.81	5.05	5.10
**LINE**	38.88	21.03	21.09
**LINE-1**	38.42	16.93	16.96
**LINE-2**	0.44	3.58	3.60
**LINE-3**	0.03	0.39	0.39
**RTE**	0.00	0.12	0.13
**ERV**	5.29	5.40	5.45
**DNA transposons**	1.98	3.00	3.03
**Total**	54.38	39.20	39.27

Table S6: Sequence characteristics of microsatellites with ≥ 15 repeat units on Y-linked scaffolds. Bases in lowercase denote regions with imperfect tandem repeats.

Scaffold	Repeat unit	Number of repeat units	Start position on scaffold	Repeat sequence (obtained from Repeatmasker)
scaffold297	G	21	23088	GGGGGGGGGGGGGGGGGGaGG
scaffold297	AG	23	27644	AGAGAGAGAGAGAGAGAGAGAGAGAGAGAGAGAGAGAGacAG AGAG
scaffold297	T	29	51610	TTTTTTTTTTTTTTTTTTTTTTTTTTTTT
scaffold297	AAAT	15	52281	AAATaaagaaAAATatAAATAAATAAATAAATAAATAAATAAATAAATA AATAAATAAATAAATAAAT
scaffold297	CTCCCTTC	49	55269	CTCCCTTCCTCCCTTCCTCCCTTCCTCCCTTCCTCCCTTCCTCCCT TCCTCCCTTCCTCCCTTCCTCCCTTCCTCCCTTCCTCCCTTCCTCC CTTCCTCCCTTCCTCCCTTCCTCCCTTCCTCCCTTCCTCCCTTCCT CCCTTCCTCCCTTCCTCCCTTCCTCCCTTCCTCCCTTCCTCCCTTC CTCCCTTCCTCCCTTCCTCCCTTCCTCCCTTCCTCCCTTCCTCCCT TCCTCCCTTCCTCCCTTCCTCCCTTCCTCCCTTCCTCCCTTCCTCC CTTCCTCCCTTCCTCCCTTCCTCCCTTCCTCCCTTCCTCCCTTCCT CCCTTCCTCCCTTCCTCCCTTCCTCCCTTCCTCCCTTCCTCCCTTC CTCCCTTCCTCCCTTCCTCCCTTCcttccttccttccttcttctttctctccttcccttcctc cctccctccctccctccct
scaffold297	GA	21	72854	GAGAGAGAGAGAGAGAGAGAGAGAGAGAGAGAGAaaGAGAGAGA
scaffold297	AGAA	16	94667	AGAAAGAAaaAGAAAGAAAGAAAGAAAGAAAGAAAGAAAGAAAGA AAGAAAGAAAGAAAGAAAGAAa
scaffold297	AC	23	192322	ACACaaACACACACACACACACACACACACACACACACACACACACA CAC
scaffold297	T	23	193512	TTTTTTTTTTTTTTTTTTTTTTT
scaffold297	CT	18	241447	CTCTCTCTCTCTcaCTCTCTCTCTCTCTCTCTCTCTCTCTc
scaffold297	T	17	254442	TTTTTTTTTTTTTTTTT
scaffold297	TTTC	24	257890	TTTCTTTCTTTCTTTCTTTCTTTCccTTTCTTTCTTTCTTTCTTTCTTT CTTTCTTTCTTTCTTTCTTTCTTTCTTTCTTTCTTTCTTTCTTTCTTTC tt
scaffold297	CA	19	259587	CACACACACACACACACACACACACACACACACACAcCACA
scaffold297	T	46	283813	TTTTcTTTTTTTTTTTTTTTTTTTTTTTTTTTTTTTTTTTTTTTTTTT
scaffold297	A	27	343300	AAAAgAAAAAAAAAAAAAAAAAAAAAA
scaffold297	A	23	344483	AAAAAAAAAAAAAAAAAAAAAAA
scaffold297	A	21	379383	AAAAAAAAAAAAAAAAAAAAA
scaffold297	T	17	388460	TTTTTTTTTTTTTTTTT
scaffold309	AC	19	23799	ACACACACACACACACACACACACACACACACACACa
scaffold309	A	24	62764	AAAAAAAAAAAAAAAAAAAAAAAA
scaffold309	ATAG	22	64857	ATAGATAGATAGATAGATAGgtagATAGATAGATAGATAGATAGATA GATAGATAGATAGATAGATAGATAGATAGATAGATAGATAGATAG
scaffold309	T	20	79617	TTTTTTTTTTTTTTTTTTTT
scaffold309	A	21	95327	AAAAAAAAAAAAAAAAAAgAA
scaffold309	T	19	109061	TTTTTTTTTTTTTTTTTTT
scaffold309	A	17	111760	AAAAAAAAAAAAAAAAA
scaffold309	T	18	116040	TTTTTTTTTTTTTTTTTT
scaffold309	A	19	127694	AAAAAAAAAAAAAAAAAAA
scaffold309	C	18	145706	CCCCCCCCCCCCCCCCCC
scaffold309	T	17	155868	TTTTTTTTTTTTTTTTT
scaffold309	A	18	159378	AAAAAAAAAAAAAAAAAA
scaffold309	T	21	185488	TTTTTTTTTTTTTTTTTTTTT
scaffold309	A	20	195689	AAAAAAAAAAAAAAAAAgAA
scaffold309	A	19	197350	AAAAAAAAAAAAAAAAAAA
scaffold309	T	22	206595	TTTTTTTTTTTTTTTTTTTTTT
scaffold309	A	29	209969	AAAAAAAAAAAAAAAAAAAAAAAAAAAAA
scaffold309	T	24	212205	TTTaTTTTTTTTTTTTTTTTTTTT
scaffold309	TG	22	285274	TGTGTGTGTGTGTGTGTGTGTGTGTGTGTGTGTGTGTGTGTGTG
scaffold309	TG	21	304646	TGTGTGTGTGTGTGTGTGTGTGTGTGTGTGTGTGTGTGTGTGt
scaffold309	T	19	307203	TTTTTTTTTTTTTTTTTTT
scaffold318	T	17	83831	TTTTTTTTTTTTTTTTT

scaffold318	A	19	110623	AAAAAAAAAAAAAAAAAAA
scaffold318	T	17	116906	TTTTTTTTTTTTTTTTT
scaffold318	A	17	127184	AAAAAAAAAAAAAAAAA
scaffold318	TG	24	201228	TGTGTGTGTGTGTGTGTGTGTGTGTGTGTGTGTGTGTGTGTGTGT<br>GTGt
scaffold318	AC	20	221123	ACACatACACACACACACACACACACACACACACACACACa
scaffold318	TA	15	231655	TATATATATATATATATATAcaTATATATATA
scaffold322	T	23	6778	TTTTTTTTTTTTTTTTTTTTTTT
scaffold322	AGAA	18	13277	AGAAAGAAAGAAAGAAAGAAAGAAAGAAAGAAAGAAAGAAAGAAA<br>GAAAGAAAGAAAGAAAGAAAGAAAGAAa
scaffold322	T	17	39419	TTTTTTTTTTTTTTTTT
scaffold322	C	19	44156	CCCCCCCCCCCCCCCCCCC
scaffold322	A	40	53605	AAAAAAAAAAAAAAAAAAAAAAAAAAAAAAAAAAAAAgAAA
scaffold322	GAA	204	104231	GAAGAAGAAGAAGAAGAAGAAGAAGAAGAAGAAGAAGAAGAAGA<br>AGAAGAAGAAGAAGAAGAAGAAGAAGAAGAAGAAGAAGAAGAAG<br>AAGAAGAAGAAGAAGAAGAAGAAGAAGAAGAAGAAGAAGAAGAA<br>GAAGAAGAAGAAGAAGAAGAAGAAGAAGAAGAAGAAGAAGAAGA<br>AGAAGAAGAAGAAGAAGAAGAAGAAGAAGAAGAAGAAGAAGAAG<br>AAGAAGAAGAAGAAGAAGAAGAAGAAGAAGAAGAAGAAGAAGAA<br>GAAGAAGAAGAAGAAGAAGAAGAAGAAGAAGAAGAAGAAGAAGA<br>AGAAGAAGAAGAAGAAGAAGAAGAAGAAGAAGAAGAAGAAGAAG<br>AAGAAGAAGAAGAAGAAGAAGAAGAAGAAGAAGAAGAAGAAGAA<br>GAAGAAGAAGAAGAAGAAGAAGAAGAAGAAGAAGAAGAAGAAGA<br>AGAAGAAGAAGAAGAAGAAGAAGAAGAAGAAGAAGAAGAAGAAG<br>AAGAAGAAGAAGAAGAAGAAGAAGAAGAAGAAGAAGAAGAAGAA<br>GAAGAAGAAGAAGAAGAAGAAGAAGAAGAAGAAGAAGAAGAAGA<br>AGAAGAAGAAGAAGAAggaGAAGAAGAAGAAGAAaaaGAAaaa<br>GAAg
scaffold322	TG	17	180871	TGTGTGTGTGTGTGTGTGTGTGTGTGTGTGttTGTGt
scaffold322	AC	17	197380	ACACACACACACACACACACACACACACACACACa
scaffold322	CA	20	212493	CACACACACACACACACACACAaaCACACACACACACACACA
scaffold322	AC	19	213052	ACACACACACACACACACACACACACACACACACACACa
scaffold322	CA	16	213587	CACAaaCACACACACACACACACACACACACA
scaffold322	CA	20	214622	CACACACACACACACACACACACACACACACACACACACAc
scaffold369	GT	23	1696	GTGTGTGTGTGTGTGTGTGTGTGTGTGTGTGTGTGTGTGTGTGTG<br>T
scaffold369	CA	25	63947	CACACACACACACACACACACACACACACACACACACACActCAtaC<br>ACACACAc
scaffold369	AC	19	64158	ACACACACACACACACACACACACACACACtcACaACACACa
scaffold369	AC	23	64880	ACACACACACACACACACACACACACACACACACACACACACACA<br>Ca
scaffold369	CA	17	65986	CACACACACACACACACACACACACACACACACA
scaffold369	CA	26	66252	CACACACACACACACACACACACACACAgaCACACACACACACAC<br>ACACACACA
scaffold369	AC	20	67968	ACACaaACACaACACACACACACACACACACACACACACAC
scaffold369	CA	20	71098	CACACACACACACACACACACACACACACACACACActCACAc
scaffold369	CA	18	72002	CACACACACACACACACACACACACACACACACACA
scaffold369	AC	18	72937	ACACACACACACACACACACACACACACACACACAC
scaffold369	CA	21	73479	CACACAcgCACACACACACACACACACACACACACACACACA
scaffold369	CA	17	75832	CACACACACACACACACACACACACACACACACAc
scaffold369	AC	16	76906	ACACgcACACACACACACACACACACACACAC
scaffold369	AC	19	78941	ACACACACACACACACACACACACACACACACACACACa
scaffold369	AC	18	80402	ACACACACACatACACACACACACACACACACaACACACa
scaffold369	AC	16	81716	ACACACACACACACACACACACACACACACAC
scaffold369	AC	22	83146	ACACACACACACACACACACatACACACACACACACACACACACAC<br>a
scaffold369	CA	24	87519	CACACACACACACACACACACACACACACACACACACACACACAC<br>ACAc
scaffold369	CA	17	88953	CACACACAgaCACACACACACACACACACACACACAc
scaffold369	CA	21	90054	CACACACACACACACACACACACACACACACACACAaCACACACAc
scaffold369	CA	17	100413	CACACACACAtaCACACACACACACACACACACACA
scaffold369	AC	17	100984	ACACACACACACACACACACACACACACACACACa
scaffold369	AC	14	101708	ACACACACACACACACACACagACACACAC
scaffold369	AC	18	103579	ACACACACACACACACACACACACACACgACACACACa
scaffold389	AC	16	7087	ACACACACACACACACACACACACACACACACa

scaffold389	TG	20	12691	TGTGTGTGtcTGTGTGTGTGtaTGTGTGTGTGTGTGTGTGTG
scaffold389	TG	20	22172	TGTGTGTGTGTGTGTGTGTGTGTGTGTGTGTGTGTGTGTGt
scaffold389	TG	21	26235	TGTGTGTGTGTGTGTGTGTGTGTGTGTGTGTGTGTGTGTGTGt
scaffold389	TG	20	52851	TGTGTGTGTGTGTGTGTGTGTGTGTGTGTGTGTGTGTGTGt
scaffold389	AC	17	73174	ACACACACACACACACACACACACACACACACAC
scaffold389	TG	18	74158	TGTGTGTGgcTGTGTGTGTGTGTGTGTGTGTGTGTGt
scaffold389	TG	19	76543	TGTGTGtagTGTGTGTGTGTGTGTGTGTGTGTGTGTGTG
scaffold393	AC	20	1822	ACACACACACACACACACACACACACACACACACACACAC
scaffold393	GT	15	13861	GTGTGTGTGTGTGTGTGTGTGTGTGTGTGT
scaffold393	CT	16	22325	CTCTCTCTCTCTCTCTCTCTCTCTCTCTCTCTc
scaffold393	GT	18	48001	GTGTGTGTGTGTGTGTGTGTGTGTGTGTGTGTGTGTg
scaffold393	CT	14	54007	CTCTttCTCTCTCTCTCTCTCTCTCTCT
scaffold393	CA	24	54039	CACACACACACACACACACACACACACACACACACAtaCACACACACA CACA
scaffold393	GT	16	61378	GTGTGTGTGTGTGTGTGTGTGTGTGTGTGTGTg
scaffold403	GT	17	16279	GTGTGTGTGTGTGTGTGTGTGTGTGTGTGTGTGTg
scaffold403	GT	19	26610	GTGTGTGTGTGTGTGTGTGTGTGTGTGTGTGTGTGTGT
scaffold403	AC	15	44337	ACACACACACACACACACACACACACACACa
scaffold403	AC	15	51140	ACACACACACACACACACACACACACACACa
scaffold420	TG	16	7834	TGTGTGTGTGTGTGTGTGTGTGTGTGTGTGTGt
scaffold420	CAA	15	29461	CAACAACAACAACAACAACAACAACAACAACAACAACAACAACAA
scaffold519	AAGG	23	1420	AAGGAAGGAAGGAAGGAAGGAAGGAAGGAAGGAAGGAAGGAAG GAAGGAAGGAAGGAAGGAAGGAAGGAAGGAAGGaaaaaagAAGG AAGGAAGGAAGG
scaffold579	T	26	10424	TTgTTTTTTTTTTTTTTTTTTTTTTTT
scaffold579	T	33	11001	TTTTTTTTTTTTTTTTTTTTTTTTTTTTTTTTT
scaffold579	AAGA	35	13351	AAGAaggaAAGAaggaAAGAAAGAAAGAaAAGAaaaAAGAAAGAAAG AagaAAGAAAGAAAGAAAGAAAGAAAGAAAGAagaAAGAAAG AAAGAAAGAAAGAAAGAAAGAAAGAAAGAAAGAAAGAAAGA AAGAAAGAAAGAtaaAAGAAAGAaa
scaffold579	A	17	14764	AAAAAAAAAAAAAAAAA
scaffold579	TC	17	16925	TCTCTCTCTCTCTCTCTCTCTCTCTCTCTCTCTC
scaffold657	AC	24	6191	ACACaaACACACACACACACACACACACACACACACACACACA CACACa

Table S7. Primer sequences and PCR conditions of fragments used for *in-vitro* validation of Y-scaffolds.

Scaffold	Forward primer (5´->3´)	Reverse primer (5´->3´)	Fragment size (bp) incl. primers	T_{A1} (°C) 14 cycles	T_{A2} (°C) 25 cycles
297	ACAGAGAGATGCGGTCCTG	GGAGACTCTGCTGACCAAGG	783	69	62
309	TCAGCCTTGCTTCATTGTTC	ATTGTGTGCCTGTTCCATTC	696	68	61
318.20	CAATGCTAGAGATGCAAGTGG	AAGCATATGAAGACACACAGTGG	757	67	60
318.21	ATGCTATGATCCAGCAATCC	TCCTTGTTAATTCTTCTCTGATGC	800	67	60
318.22	CTGACATACGTGGCTCACAG	ATGTGGAGGTTCAGGAGGAC	800	67	60
318.23	GTGGTCAGATCCAGCTCCTC	GACCTTGCTTCTGCCTTCTC	786	67	60
*322***	GAGTAGAGCTGGTGCTTGTGAG	GAAGCAGAGCTCAAGTCTGAAG	687	68	61
369	CATTGAAACAAGGGCACATC	TCCTCTAGGAACCCAACTGC	780	68	61
393	AATTGTGAAGGATGAGGAAGG	CCAGAGAAGCAGAGGATGG	788	70	63
*389***	ACCCACTGCTGTTCTGTATCC	CCAACAGTGTAGTGGTTGTGC	679	68	61
*403***	CACTCAGGAGAGCACAGGTC	TGTGTGTCGTAAGCAGAGGTC	796	68	61
420	TTCAGAGGGAAGGGTGTAGG	AATGGTAGTGAGGGAGGTATGG	635	69	62
519	CCCAAGTTAGGGAAGTTTGC	TGTTTCAGCTTCAGCTGTCC	734	68	61

*579**	CTGCAGGCCTGTCAATGTTA	TGTGTATCGACCCCATACTTTG	660	66	61
605	TTTGACGGCTGAGCAATATC	GATGCAGCCACAATGAAATC	706	68	61
646	TGTCTAGCCATCTGGTCCTG	CATGGACATTGTTGCATTGA	778	69	62
657	TCCAGGTTGTCAAGCACATG	CTTGCTCCATCCACATGCTG	769	69	62
596	ATCACCCTCTCCACTCACAA	CCACCTCTTTGACTTCCTGG	735	69	62
613	TTGTGGACATTGCTGCTTCT	TCTGAACATAGGCTGCAACC	674	69	62
632	CTATCGTGGACATTGCTGCT	CTATCGTGGACATTGCTGCT	621	69	62
771	AGAACTTCCTGTACCTGGCT	GCTGATTCCGTGGATGTTCA	753	69	62
795	GGAACACGACAAGGATCTCC	TTGTGCCAATACCATGCTGT	705	69	62
813	ATCTTGCTGCTCCTCTGTTG	CCACGTCACATCATACTGCA	767	69	62
236a	GACTCAAGCTCAGCGTTCAC	GTCCAGTGGAAGAGGTAGGC	760	68	61
267a	ATACATACGCGCACACACAC	GCACATACCTCGTCAAGGAC	655	68	61
253x	CTGGAGGTCTGTGTGTGGAG	AACCTAATGCGTGTGTCTGC	796	68	61
301x	TGGATAAGGCATTCTGCAAG	CTGAAGGCAGTGGTGAAGAG	717	68	61

* Primers from (Bidon et al. 2014) (fragment *579.1B*)
** Primers from (Kutschera et al. 2014)
a Autosomal scaffold based on AD-ratio
x X-linked scaffold based on AD-ratio

Table S8. Accession numbers and sample origin of polar, brown and black bear genomes

Species	Sample origin	Number of individuals	Accession numbers
Ursus maritimus	Spitsbergen, Svalbard	9	SRX155945, SRX155949, SRX155951, SRX155953, SRX155954, SRX155955, SRX155957, SRX155960, SRX155961
	Alaska	3	SRX156102, SRX156103, SRX156105
Ursus arctos	ABC-Islands	1	SRX156108
Ursus americanus	Alaska	1	SRX156137
